# The earliest ossicone and post-cranial record of *Giraffa*

**DOI:** 10.1371/journal.pone.0185139

**Published:** 2017-09-19

**Authors:** Melinda Danowitz, John C. Barry, Nikos Solounias

**Affiliations:** 1 Department of Anatomy, New York Institute of Technology College of Osteopathic Medicine, Old Westbury, NY, United States of America; 2 Department of Pediatrics, A.I. DuPont Hospital for Children, Wilmington, DE, United States of America; 3 Department of Human Evolutionary Biology, Peabody Museum, Harvard, Cambridge, MA, United States of America; 4 Department of Paleontology, American Museum of Natural History, New York, NY, United States of America; Medical University of South Carolina, UNITED STATES

## Abstract

The oldest *Giraffa* material presently known consists of dental specimens. The oldest post-cranial *Giraffa* material belongs to the Plio-Pleistocene taxon *Giraffa sivalensis*, where the holotype is a third cervical vertebra. We describe three non-dental specimens from the Early Late Miocene of the Potwar Plateau, including an 8.1 million year old ossicone, 9.4 million year old astragalus, and 8.9 million year old metatarsal and refer them to *Giraffa*. The described ossicone exhibits remarkable similarities with the ossicones of a juvenile modern giraffe, including the distribution of secondary bone growth, posterior curvature, and concave pitted undersurface where the ossicone would attach to the skull. The astragalus has a notably flat grove of the trochlea, medial twisting between the trochlea and the head, and a square-shaped sustentacular facet, all of which characterize the astragalus of *Giraffa camelopardalis*. The newly described astragalus is narrow and rectangular, unlike the boxy shaped bone of the modern giraffe. The metatarsal is large in size and has a shallow central trough created by thin medial and lateral ridges, a feature unique to *Giraffa* and *Sivatherium*. Our described material introduce the earliest non-dental material of *Giraffa*, a genus whose extinct representation is otherwise dominated by teeth, and demonstrate that the genus has been morphologically consistent over 9 million years.

## Introduction

*Giraffa* is currently represented by four extant species that are found throughout Africa [1]. These distinct species have been separated using differences in the nucelear and mitochondrial genetic makeup, however the skeletal and soft anatomy of the four taxa has yet to be studied and described. The oldest representatives of this genus include *Giraffa priscilla* and *Giraffa punjabiensis*, taxa that are known from the Middle and Lower Siwaliks [2–4]. *Giraffa priscilla* and *Giraffa punjabiensis* have been named based on teeth, and subsequent studies have referred additional dental specimens to these taxa [5–11]. Younger *Giraffa* species are known from the Pliocene and the Plio-Pleistocene, including *Giraffa sivalensis* from the Upper Siwaliks [2,12], and *Giraffa jumae* and *Giraffa stillei* from localities in East Africa [13]. Our study introduces for the first time post-cranial material of *Giraffa* from the Middle Siwaliks, providing the oldest known non-dental specimens of this genus.

While there are currently only two extant genera of Giraffidae, the okapi (*Okapia*) and the giraffe (*Giraffa*), the Late and Middle Miocene sampled a more diverse array of giraffids throughout Africa, Europe and Asia. Among these, *Samotherium major* and *Giraffokeryx punjabiensis* are the better known. Although sampled from the Late and Middle Miocene of the Potwar Plateau, *Giraffa punjabiensis* and *Giraffa priscilla* are less well known. Dental material from *Giraffa punjabiensis* has been reported along with sivatheres from the Dhok Pathan Formation [3], which ranges from 10.1–3.5 Ma [14]. *Giraffa priscilla*, a slightly older taxon, has been reported along with the better known giraffid *Giraffokeryx punjabiensis* from the Chinji Formation [2], which ranges from 14–10.8 Ma [15].

*Giraffa sivalensis* is a geologically younger species of *Giraffa* known from the Upper Siwaliks [2]. This was the first extinct *Giraffa* species found, and unlike *Giraffa priscilla* and *Giraffa punjabiensis* where the type specimens are dentitions, the holotype of *Giraffa sivalensis* is a third cervical vertebra [12]. *Giraffa sivalensis* is based on both dental and post-cranial material; the skeletal specimens allow for hypotheses regarding neck elongation and general body and metapodial size. Features of the third cervical vertebra support the theory that *Giraffa sivalensis* is transitional in the elongation of the posterior portion of the vertebra, the stage responsible for the extreme elongation that characterizes the neck of the modern giraffe [16]. Body size analyses incorporating the neck, teeth, and limbs also allow for the evaluation of potential sexual dimorphism within this taxon [17]. We describe the oldest post-cranial specimens and only non-dental material from the Miocene of *Giraffa*, allowing for a broader understanding of the genus.

## Material and methods

### *Giraffa* spp.

Complete detached left ossicone: YGSP 16274

Slightly damaged left astragalus: YGSP 51835

Fragmentary metatarsal specimen: YGSP 14906a and YGSP 14906b

### Giraffa camelopardalis

Juvenile Ossicone (detached from skull): AMNH 83605, AMNH 165052, AMNH 53546

Young Adult Ossicone (fused to skull with visible fusion line): AMNH 165051, AMNH 14135

Astragalus: AMNH 82001, AMNH 82003, AMNH 83458, AMNH 53550

Metatarsal: AMNH 53543, AMNH 82001, AMNH 14135, AMNH 53550, AMNH 70016.

We provide detailed descriptions of three specimens on loan from the Geological Survey of Pakistan, currently housed at Harvard. We refer these specimens to *Giraffa* spp. based on the notable similarities with equivalent specimens of *Giraffa camelopardalis*. For comparison, we describe the morphology of the ossicone (juvenile and young adult stages), astragalus, and metatarsal of *Giraffa camelopardalis* based on specimens at the American Museum of Natural History. All specimens were collected legally and have been housed in established natural history museums. All specimens are accessible to visiting scientists with permission from the curators.

The ages of localities and fossils were determined by collecting samples for magnetostratigraphy and radiometric dating. The specific methods of determination of the ages of these deposits have been discussed extensively [14,18]. The researchers measured 20 rock sections that rage in thickness between 250 and 3200 meters. They also measured 27 shorter sections. In many sections continuous tracing of horizons was possible. In other cases correlations were accomplished by correlations of the magnetic-polarity stratigraphy. No sections were correlated on the basis of biostratigraphy.

The Siwalik specimens were found during a series of field expeditions that were conducted between Harvard University and the Geological Survey of Pakistan (GSP) in the Siwalik deposits of the Potwar Plateau from 1981 to 1995. These expeditions were led by a large team of collaborators that included researchers from Peshawar University, Dartmouth College, Lamont-Doherty Geological Observatory, Department of Geosciences of the University of Arizona, Museum of Paleontology University of Michigan, and the Department of Paleobiology at the Smithsonian. The *Giraffa* material, as well as many other fossil specimens were found by members of the GSP-Harvard research group in 1981. At that time, the field research was supported by the NSF and Smithsonian Foreign Currency Program grants to David Pilbeam who was the head organizer.

### Institutional abbreviations

YGSP, Geological Survey of Pakistan, Islamabad, Pakistan; AMNH, American Museum of Natural History, New York, USA.

## Description of new *Giraffa* material

Left Ossicone

Age: 8.1 Ma

**Fig 1:**
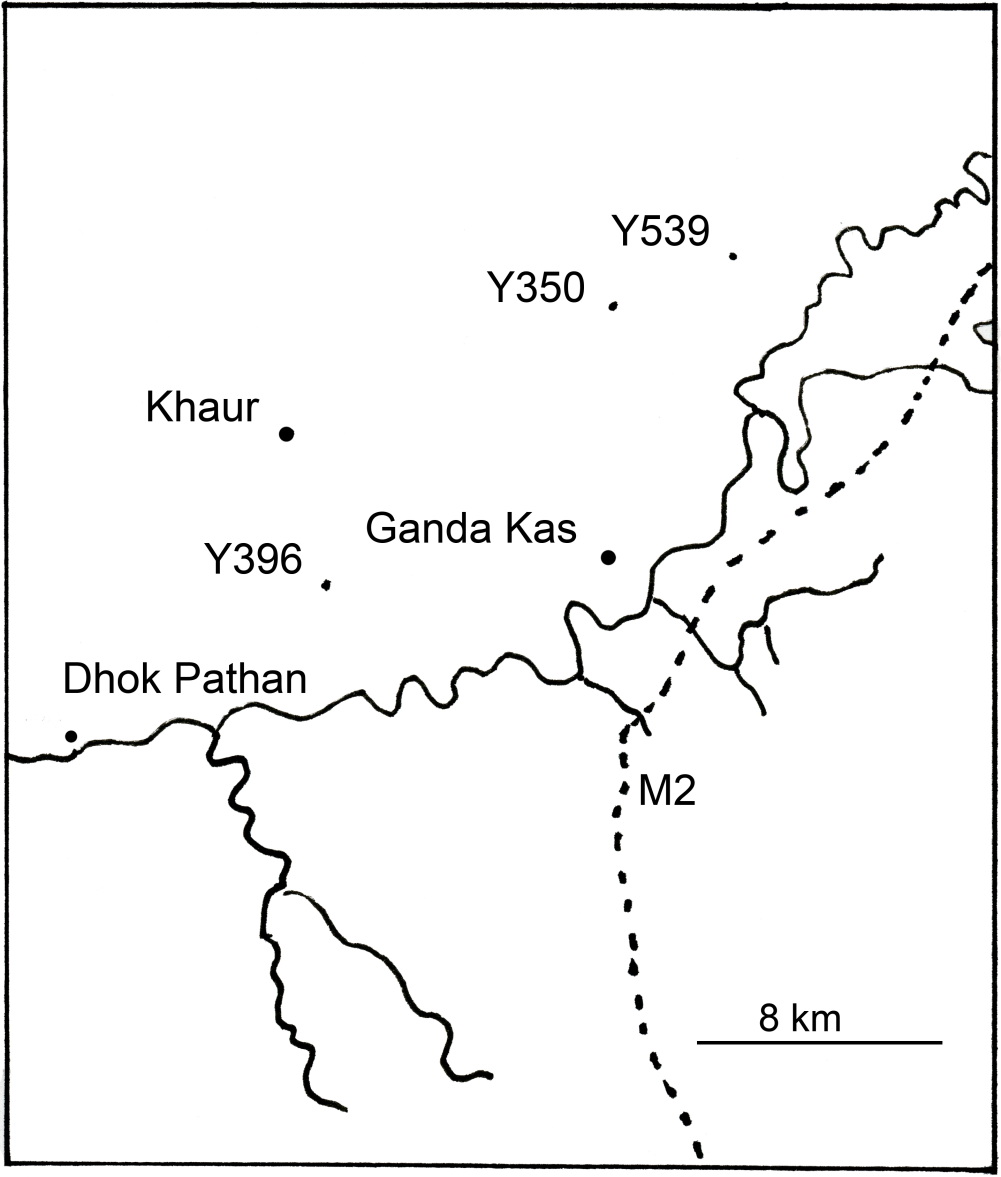
Map of the region where the three localities are located. This region is near Khaur and Ganda Kas, two villages of the Potwar Plateau in Pakistan.

Locality: Y539 (Latitude: 33^o^ 18’ 50.46” N, Longitude: 72^o^ 41’ 13.44” E) (Fig 1)

**Fig 2:**
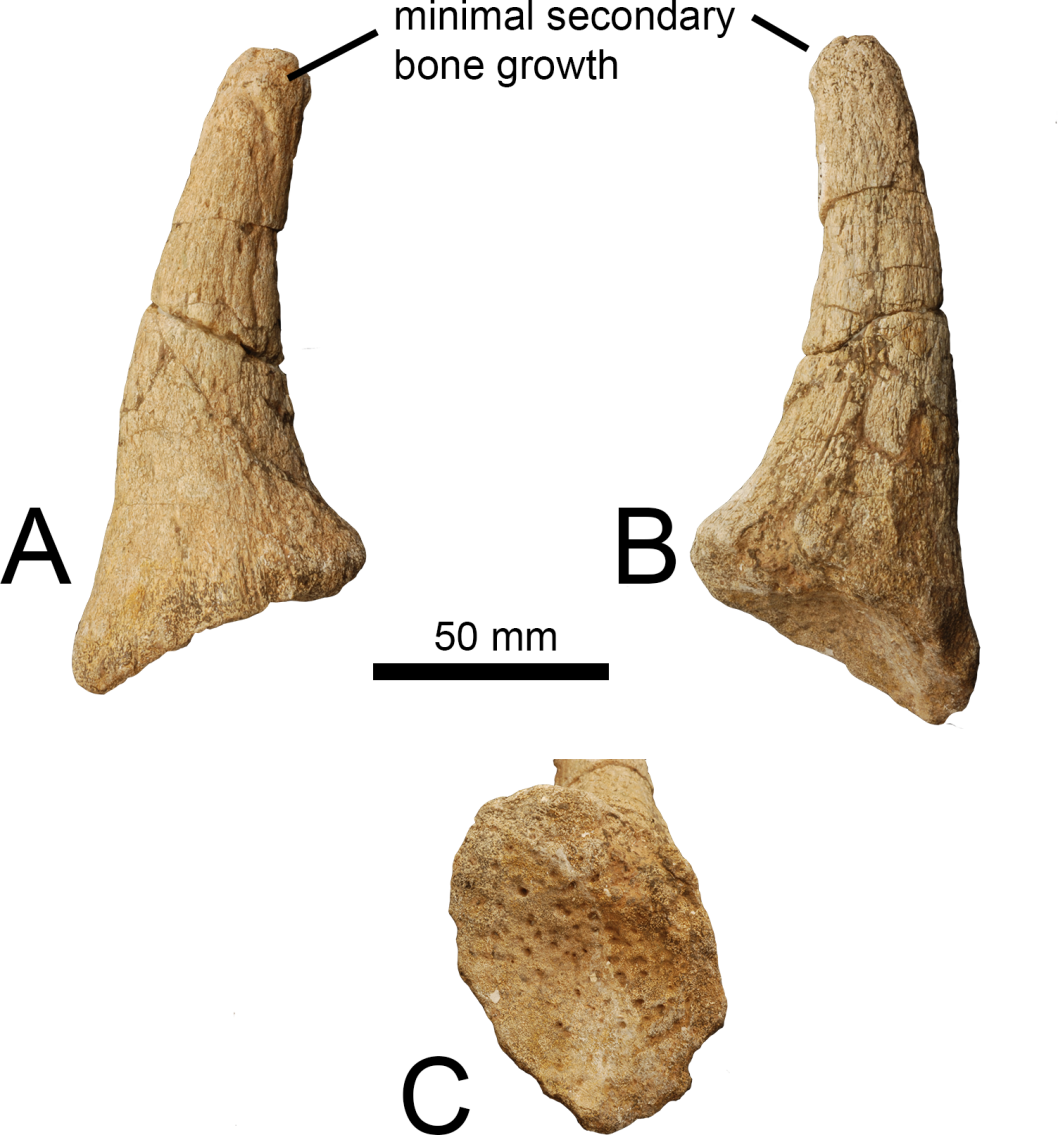
Ossicone of *Giraffa* sp. (A) *Giraffa* sp. ossicone (YGSP 16274) in lateral view. (B) *Giraffa* sp. ossicone in medial view. (C) Undersurface of *Giraffa* sp. ossicone. It is likely that this ossicone belonged to a young adult individual, as the undersurface exhibits the characteristic pitting seen in ossicones not yet fused to the skull.

This is an ossicone likely belonging to a young individual as it is detached; the undersurface exhibits the characteristic pitting seen when the ossicone is not yet fused to the skull roof. The bone between the individual pits is smooth. The entire surface of the ossicone has fine lineations, which are present along the length of the shaft but are not continuous longitudinally. The anterior surface exhibits several elongated lumps of secondary bone growth that appear to descend from the apex. The lateral, medial, and posterior surfaces are relatively smooth, although the medial surface is weathered. The secondary bone growth is concentrated at the apex, where it presents as lumps of bony material. There are small foramina scattered over the lateral shaft and apex. The under-side is concave, implying that the ossicone sat upon a small boss on the skull. The shaft is curved posteriorly and is oval in cross section towards the base and becomes more circular towards the apex. The base is broader than the ossicone shaft. The apex is of equal width to the distal shaft and is rounded. There are several bulges of secondary bone growth at the apical tip (Fig 2).

### Measurements

Length x width of ossicone 30 mm above base: 50 mm x 35 mm

Length x width of ossicone at apex: 21 mm x 22 mm

Axial length of ossicone: 130 mm

### Left astragalus

Age: 9.4 Ma

Locality: Y350 (Latitude: 33^o^ 18’ 13.98” N, Longitude: 72^o^ 38’ 49.50” E) (Fig 1)

**Fig 3:**
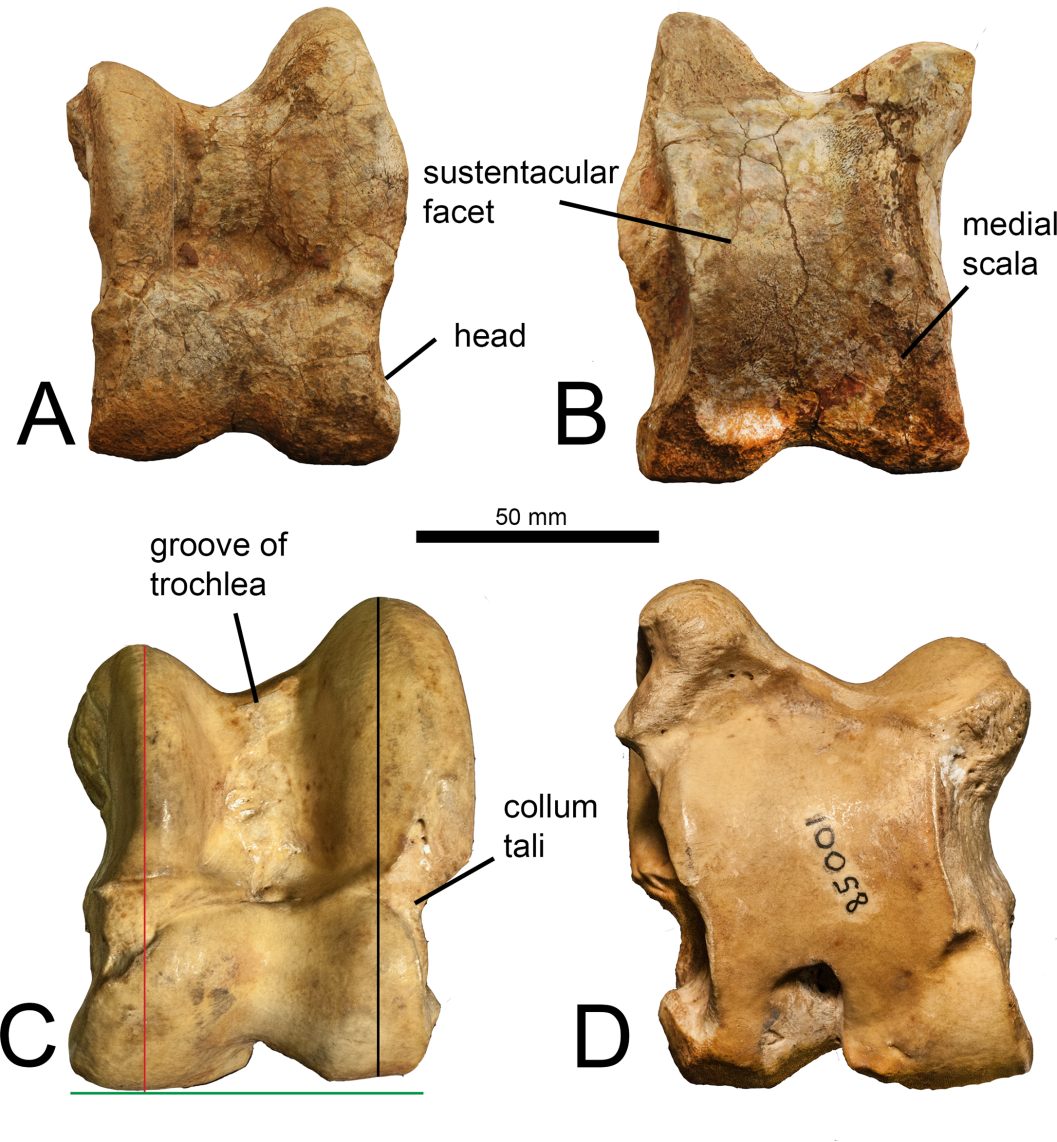
Astragali of *Giraffa* sp. and *Giraffa camelopardalis*. (A) *Giraffa* sp. left astragalus (YGSP 51835) in dorsal view. (B) *Giraffa* sp. astragalus in ventral view. (C) *Giraffa camelopardalis* right astragalus (AMNH 82001) in ventral view. (D) *Giraffa camelopardalis* astragalus in dorsal view. Proximodistal medial length is shown in red, proximodistal lateral length is shown in black, and distal width is shown in green. Note: *Giraffa camelopardalis* astragalus is reversed to facilitate comparisons.

In dorsal view, the lateral edge of the trochlea is notably taller than the medial edge. The lateral edge of the trochlea is curved medially, creating a cocked appearance of the astragalus. The groove of the trochlea is exceptionally flat. The central fossa is large and deep. The trochlea is twisted in relation to the head of the astragalus, so that the medial surface is visible in dorsal view. The proximal edge of the articular surface of the head has a central depression, and is vertical laterally and deeply slanted medially. The medial aspect of the head is significantly larger than the lateral aspect. The collum tali is short and is wider on the lateral edge. There is a distinct bulge at the medial collum tali that is pointed, and a rounded bulge at the lateral collum tali. There is a deep notch on the lateral edge of the astragalus, between the trochlea and the head. The distal notch on the distal aspect of the head is large, creating a distinct lateral and medial bulge. The astragalus is rectangular-shaped. In ventral view, the interarticular groove is not continuous with the proximal triangular fossa. There is a slight notch on the lateral aspect of the proximal-lateral trochlea. Although fragmented, it is evident that the medial ridge is oriented vertically towards the medial trochlea, creating a square shaped ventral articular surface, or sustentacular facet. There is a small and faint medial scala. There is a wide depression at the distal, lateral edge of the ventral articular surface. In medial view, there is an exceptionally deep pit at the head. The proximal groove for the tibia is shallow and notably wide. In lateral view, the proximal protrusion that articulates with the fibula is notably large and pointed. This connects to a short ridge that extends midway up the lateral trochlea. There is an expansive fossa that comprises the distal half of the lateral surface, excluding the distal facet for the calcanuem. The distal facet for the calcaneum is small and oval shaped. The lateral surface of the trochlea is rounded (Fig 3).

### Measurements

Lateral proximodistal length: 96 mm

Medial proximodistal length: 81.3 mm

Distal width: 60.8 mm

### Midshaft metatarsal fragments

Age: 8.9 Ma

Locality: Y0396 (Latitude: 33^o^ 10’ 59.43” N, Longitude: 72^o^ 30’ 46.18” E) (Fig 1)

**Fig 4:**
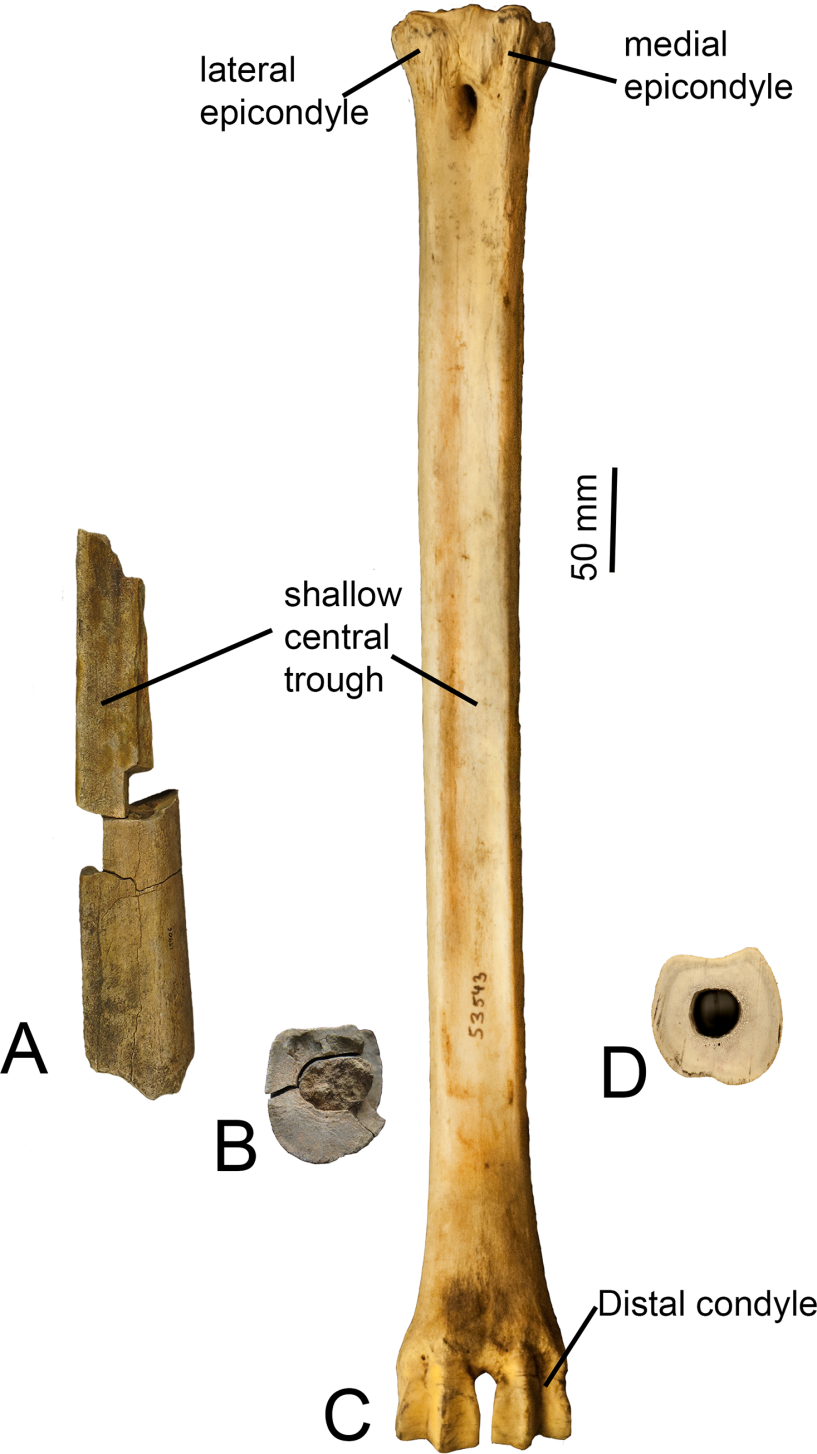
Metatarsal plantar and cross-sectional views. (A) *Giraffa* sp. (YGSP 14906a and YGSP 1906b) metatarsal. (B) Cross section of distal end of *Giraffa* sp. metatarsal. (C) *Giraffa camelopardalis* (AMNH 53543) complete metatarsal. (D) Cross section towards distal shaft of *Giraffa camelopardalis* metatarsal (NS 289). The central trough of both metatarsals is notably shallow.

These are two fragments from the same individual. One fragment preserves about 15 cm of the length and the entire cross section of the shaft, whereas the other fragment is only the plantar surface of the shaft. The central trough of the plantar surface is exceptionally shallow in depth. There are two faint ridges, one medially and one laterally. One ridge is slightly more elevated and sharp, and the other ridge is more rounded. There is a faint pyramidal rise at presumably the distal end of the shaft fragment. There is a faint dorsal longitudinal sulcus on the dorsal surface. The central canal does not have a septum. The homogeneity of width of the two fragments clearly indicates that the entire bone was elongated (Fig 4).

### Measurements

Dorsoplantar height at distal break: 43 mm

Mediolateral width at distal break: 37 mm

## Description of *Giraffa camelopardalis* material

### Juvenile ossicone

**Fig 5:**
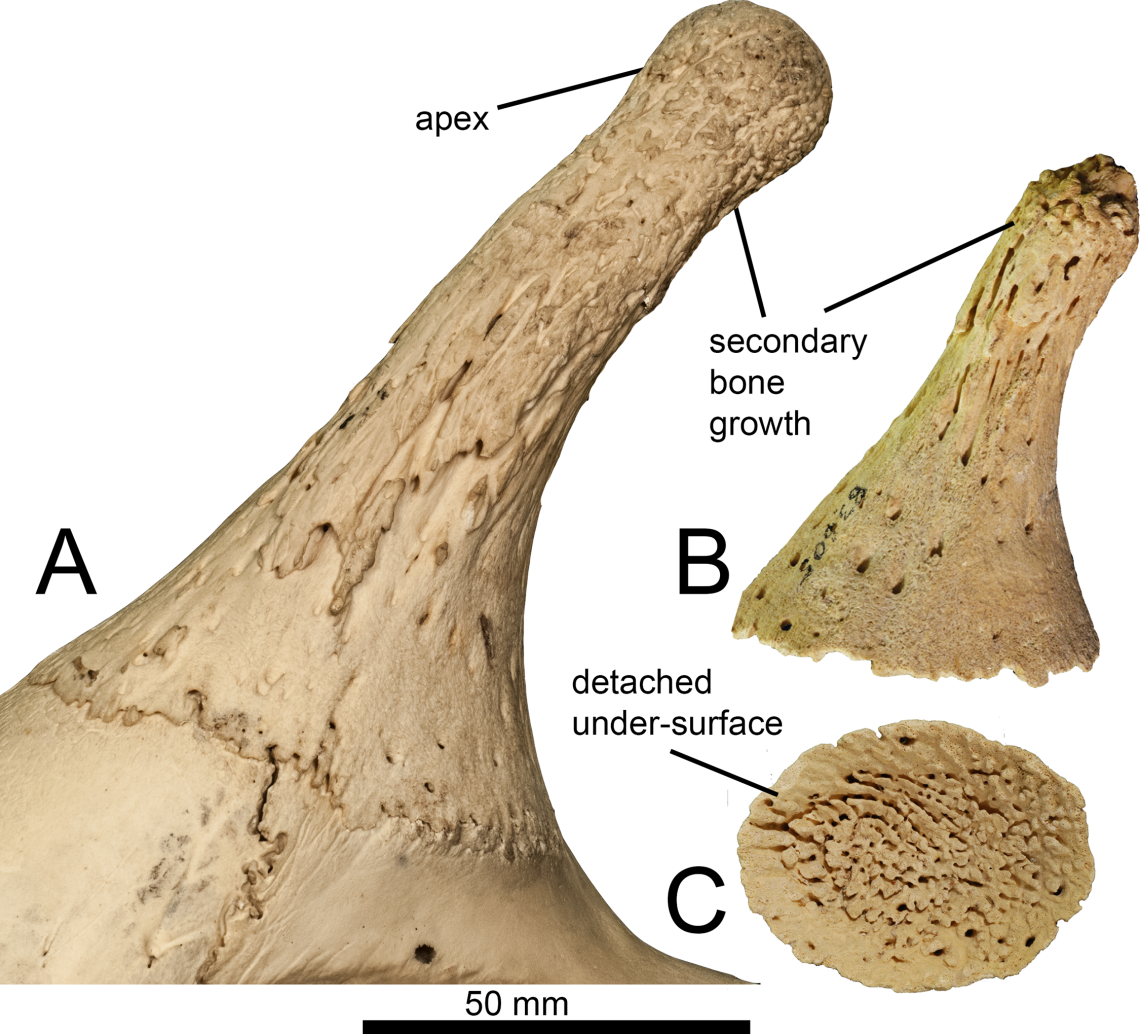
Juvenile and young adult ossicones of *Giraffa camelopardalis*. (A) Lateral view of *Giraffa camelopardalis* young adult female ossicone (AMNH 165051), with secondary bone growth concentrated at the apex and as irregularly bordered islands on the shaft. The ossicone is fused to the skull surface with a visible fusion line. (B) Lateral view of *Giraffa camelopardalis* juvenile ossicone (AMNH 83605) that is not yet fused to the skull. (C) Undersurface of *Giraffa camelopardalis* juvenile ossicone, showing the characteristic pitted surface of detached ossicones.

The undersurface of the ossicone is oval-shaped and has numerous pits. The pits are more concentrated centrally, and the outer rim of the undersurface is smooth. The ossicone surface has lineations that are most pronounced distally towards the apex, whereas the base of the ossicone is smoother. The lineations do not continue down the entire length of the shaft. There is secondary bone growth concentrated at the apex that presents as elongated oval shaped lumps. There are numerous large foramina throughout the base, shaft, and apex. The under-side of the ossicone is concave, which corresponds to the domed portion of the skull upon which the ossicone sits. The ossicone shaft is curved posteriorly and is oval-shaped in cross section towards the base and is more circular in cross section towards the apex. The apical width is equal to the width of the distal shaft. The apex is rounded with small knobby protrusions of secondary bone growth at the tip (Fig 5).

### Young adult ossicone

The ossicone is fused to the skull and is positioned posterior to the orbit. There is a distinct line of fusion between the base and the skull surface. There is abundant secondary bone growth that appears to descend from the apex down the shaft. The secondary bone growth presents as flattened islands with irregular borders that are distinct from the ossicone shaft. The secondary bone growth is most concentrated at the apex and on the anterior surface of the shaft. There are small foramina scattered on the shaft and concentrated circumferentially around the base. The ossicone is curved posteriorly. The base is broader than the ossicone shaft. The apex is slightly wider than the distal shaft. The apex is rounded and has small circular lumps of secondary bone growth (Fig 5).

### Astragalus

The astragalus is notably wide and boxy shaped. In dorsal view, the lateral trochlea is massive and the lateral edge is curved, creating an overall cocked appearance. The groove of the trochlea is wide and exceptionally flat, and it continues distally into the central fossa. The trochlea and the head are twisted, so that the medial astragalar edge is partially visible in dorsal view. The proximal articular surface of the head has a central depression with a vertical lateral edge and slanted medial edge. The collum tali is tall. The astragalus is notched laterally between the trochlea and the head. There is a distinct separation between the medial and lateral aspects of the head. Ventrally on the astragalus, the sustentacular facet is full and square shaped. The medial scala is faint, and the distal intracephalic fossa is flat and pronounced (Fig 3).

Average lateral proximodistal length: 91.2 mm

Average medial proximodistal length: 81.5 mm

Average distal width: 65.4 mm

See Solounias and Danowitz (2016) for a complete astragalar description and measurements of *Giraffa camelopardalis* and other giraffids [19].

### Metatarsal

The medial and lateral epicondyles are symmetrical. The lateral epicondyle is separated into a plantar and dorsal head by a deep obliquely oriented groove. The medial epicondyle is separated into a plantar and dorsal head by a shallow groove. Both heads of both epicondyles flare outward, more notably on the dorsal heads. There is no distinct pygmaios at the proximal surface. There is an oval shaped thin protrusion centrally on the medial epicondyle. The medial and lateral ridges are thin and create a shallow central trough. The central trough further flattens at the distal shaft, and in this area, there is a small pyramidal rise. The distal shaft flares medially towards the distal condyle (Fig 4).

Mediolateral width at diaphysis: 46.32 mm

See Rios et al. (2016) for a complete metatarsal description and measurements of *Giraffa camelopardalis* and other giraffids [20].

## Discussion

The ossicone of the modern giraffe forms as an initially detachable dense connective tissue growth that subsequently fuses to the skull roof [21–23]. This fusion begins to occur around age 6 [23]. The undersurface of the described *Giraffa* sp. ossicone exhibits the characteristic pitting seen when the cranial appendage is not yet fused to the skull; the individual was presumably a juvenile. It is also possible that the individual was a young adult. It is not possible to definitively determine if this ossicone belonged to a male or female individual. This ossicone is exceptionally similar to that of a young *Giraffa camelopardalis* individual, notably in the shape of the shaft that is curved posteriorly, oval in cross section, and rounded at the apex. In a typical modern giraffe, secondary bone growth is widespread and often irregularly distributed on the ossicone and extends onto the skull roof [21, 24–25]. In the described specimen of *Giraffa* sp., the secondary bone growth is concentrated at the apex, and in elongated lumps on the anterior shaft. The relative lack of secondary bone growth in the extinct taxon may reflect the younger age of the individual, supported by the pitted undersurface, or may represent interspecific differences and/or sexual dimorphism among *Giraffa* species.

*Bohlinia attica* is commonly referred to as an extinct close relative to the modern giraffe based on the elongated C2, C7, which are the only known vertebrae, and metapodials [16,20,26]. The ossicone, however, has features notably different from both the giraffe and the described *Giraffa* sp. ossicone. The ossicone of *Bohlinia attica* is large; the base of the ossicone comprises approximately ¼ the length of the cranium [27]. The ossicone is also conical, compressed, and rectangular in cross-section, unlike the elongated, rounded, and oval in cross-section ossicone characteristic of both *Giraffa* species. In addition, the *Giraffa* ossicones have characteristic lumps of secondary bone growth concentrated at the apex, and in older individuals, the secondary bone growth expands to cover the shaft and parts of the cranium. In *Bohlinia attica*, the secondary bone growth presents as linear streaks on the shaft, seen frequently in sivatheres [27]. There are several post-cranial differences between the giraffe and *Bohlinia attica* as well; in *Bohlinia*, the ulna is more reduced, the calcaneum is more elongated, and the pyramidal exhibits straighter proximal articular surface [28]. The shape, distribution, and overall structure of the ossicone are notably similar in *Giraffa* sp. and *Giraffa camelopardalis*, despite an 8 million year age gap, and are markedly different from the closely related *Bohlinia attica*.

Astragalar morphology provides information about both the paleohabitat as well as the phylogeny of an individual [29–32]. However, in our study we are not addressing paleoecological implications of the single astragalus. Anatomical features of the giraffid astragalus do allow for the diagnosis and identification of species based on unique combinations of characteristics [19]. The *Giraffa* sp. astragalus shares many similarities with that of the modern giraffe. Both *Giraffa* species share a curved lateral trochlear edge, creating a “cocked” appearance, a slight medial twisting of the trochlea in relation to the head, a square-shaped sustentacular facet, and a central depression at the proximal edge of the articular surface of the head. In addition, the *Giraffa* sp. astragalus exhibits an exceptionally flat groove of the trochlea, a feature shared only with the two extant giraffid species. Several features, however, distinguish the *Giraffa* sp. astragalus from the modern giraffe. In *Giraffa* sp., the collum tali is short, whereas it is exceptionally tall in the giraffe, and there is a large fossa on the distal lateral surface, which is absent in the giraffe.

The *Giraffa* sp. astragalus is rectangular-shaped, similar to the majority of giraffids. The *Giraffa* sp. specimen is different from the modern giraffe, *Sivatherium*, *Helladotherium*, *Honanotherium*, and *Bohlinia*, in which the astragalus is boxy and wide [19]. We believe this wider-than-long shape relates to the large body size of these giraffids, and that a relatively wider astragalus can better accommodate substantial body weight. While this shape difference might reflect phylogeny or paleohabitat, these taxa belong to three different subfamilies, and derive from ecologies ranging from savannah to dense forests, making these associations with astragalar shape less likely [33]. *Giraffa* sp. exhibits a rectangular-shaped astragalus, suggesting this might have been a smaller taxon.

Although the *Giraffa* sp. metatarsal specimens are fragmented, they share many similarities with the metatarsals of *Giraffa camelopardalis*. In both taxa, the central trough is exceptionally flattened. This feature is unique to *Giraffa* and *Sivatherium*; the trough of all other giraffids is intermediate or deep [20]. *Sivatherium* is significantly larger than all other giraffids, and unlike the metatarsals of *Giraffa* sp. and *Giraffa camelopardalis*, the *Sivatherium* metapodials are short and robust [20,34]. The central trough of *Giraffa camelopardalis* shows variation; in the majority of specimens, there is a very slight trough, however in some individuals, the trough is completely absent, or very rarely, the trough is deeper. This variability on the trough of the giraffe may represent intraspecific or locomotory differences, as the giraffe has a wide geographic range. This might also represent interspecific differences, however the skeletal morphologies of the newly identified four species of the modern giraffe [1] have yet to be described. The variability might also be related to the age, body size, and gender of the individual. The *Giraffa* sp. and *Giraffa camelopardalis* metatarsals appear to be similar in height and width, evidenced by the comparably sized cross sections.

Interestingly, although the *Giraffa* spp. specimens are 8–9 million years older than the modern giraffe, we find very few differences in the skeletal features. Although difficult to compare since equivalent specimens are lacking, our described post-cranial specimens generally appear to be more similar to *Giraffa camelopardalis* than those of *Giraffa sivalensis*, a species that is geologically younger and closer in age to the modern giraffe. *Giraffa sivalensis* exhibits cervical characteristics similar to *Giraffa camelopardalis*, such as a posteriorly directed C3 spinous process, lack of laminar ridges, and reduced ventral notch, indicating that both taxa exhibit some degree of caudal vertebral elongation [16]. However, the C3 holotype of *Giraffa sivalensis* is smaller than that of the modern giraffe, and the caudal portion of the bone comprises a smaller proportion of the total vertebral length than that of *Giraffa camelopardalis*. The limbs of *Giraffa sivalensis* exhibit a deep central trough, a feature more similar to that of *Bohlinia*, *Helladotherium*, and *Birgerbohlinia* [20].

In the absence of further material, we are unable to determine if the three described *Giraffa* specimens belong to one or multiple species. Since the two known Miocene species, *Giraffa punjabiensis* and *Giraffa priscilla*, are only identified by isolated dentitions, we cannot link the post-cranial material to either species at this time. Research shows that the longevity of a species or genus of artiodactyls averages around 3 million years [35]. It is therefore possible that our three specimens belong to a single *Giraffa* species.

The new post-cranial material referred to *Giraffa* indicates that the skeletal morphology of the genus has been relatively consistent over the past 9 million years. The astragalus is the oldest of the three specimens and it differs in overall astragalar shape, but still exhibits morphological similarities including the flattened trochlear groove, lateral notch, square shaped sustentacular facet, and medial twisting between the head and trochlea. The metatarsals of both *Giraffa* sp. and *Giraffa camelopardalis* have a flattened central trough, a feature rare among giraffids. In addition, the ossicone of *Giraffa* sp. is remarkably similar to that of young modern giraffe individuals. The similarities between *Giraffa* spp. and *Giraffa camelopardalis* reinforce the notion that *Giraffa* evolved in the Indian Subcontinent in the Late Miocene and migrated later to Africa. The described specimens are the first and only Miocene non dental material of *Giraffa* known, and are pivotal in our understanding of the ever-surprising genus.
